# Effect of dietary cation–anion difference on dry matter intake, digestibility, body weight gain, blood parameters, and carcass traits in Zandi lambs

**DOI:** 10.1093/tas/txad019

**Published:** 2023-03-03

**Authors:** Mohammad Khani, Amir Fattah, Sayyed R Ebrahimi-Mahmoudabad, Sahereh Joezy-Shekalgorabi

**Affiliations:** Department of Animal Science, Faculty of Agriculture, Shahr-e Qods Branch, Islamic Azad University, Tehran, Iran; Department of Animal Science, Faculty of Agriculture, Shahr-e Qods Branch, Islamic Azad University, Tehran, Iran; Department of Animal Science, Faculty of Agriculture, Shahr-e Qods Branch, Islamic Azad University, Tehran, Iran; Department of Animal Science, Faculty of Agriculture, Shahr-e Qods Branch, Islamic Azad University, Tehran, Iran

**Keywords:** carcass quality, dietary cation–anion difference, digestion, heat stress, weight gain, Zandi sheep

## Abstract

The dietary cation–anion difference (DCAD) has gotten much attention recently; however, there is not much evidence on organic matter digestibility, blood parameters, dry matter intake, body weight, and carcass features of male sheep fed with different DCAD diets. The effects of dietary cation–anion difference (DCAD) on these traits in male lambs under the environmental high temperatures were investigated in this study. Forty male lambs (average body weight of 39 kg) were randomly assigned to one of five treatments with eight replicates. Lambs were fed diets with DCAD levels ranging from 150 (control group) to 300, 450, 600, and 750 mEq/kg dry matter. This study lasted 100 d and used a 21-d adaptation. The results showed that the control group had the highest dry matter intake, dry matter digestibility, and crude protein digestibility (*P* = 0.02). Also, the lowest amount of average body weight was observed in the control group (*P* = 0.01). The results showed the different DCAD levels affected the statistical significance in terms of live weight, carcass weight, length and width of muscle cross section, lung weight, spleen weight, and abdominal fat (*P* = 0.04). As well, the highest ruminal pH was observed in the control group (*P* = 0.4). The results of the blood glucose parameter showed that control group had a significant effect on the blood glucose level (*P* = 0.04). Furthermore, the highest abdominal fat weight was observed in the control group (*P* = 0.04). There was no statistically significant difference between other traits, including skin weight, head weight, leg weight, carcass length, liver weight, kidney weight, heart weight, testicle weight, tail weight, rumen weight, and lactation weight. In summary, increasing DCAD in the diet could improve the production and carcass quality in lambs under environmental high temperatures.

## INTRODUCTION

The cation–anion difference (DCAD) is defined in its most basic form as the difference in concentrations of the major anions (Cl− and S2−) and cations (Na+ and K+) per kilogram of diet dry matter (Block, 1984). Tucker et al. (1988) define the cation–anion difference (DCAD) as the potential positive or negative change caused by nonmetabolizable dietary ion mixtures. Block (1984) demonstrated the initial benefit of feeding a different DCAD by observing that treatment with −172.3 (mEq/kg DM) DCAD could prevent hypocalcemia compared to a control DCAD of +448.6 (mEq/kg DM). Investigations have revealed that diets with a lower DCAD could improve health and develop the economic life of livestock animals (Leno et al., 2017; Melendez et al., 2017; Al-Rabadi et al., 2018). Lately, attention has been given to the interaction of DCAD with vitamin D (Rodney et al., 2018a; Martinez et al. 2018a, 2018b), 5-hydroxyl-tryptophan (Slater et al., 2018), cholecalciferol/calcidiol (Rodney et al., 2018b), calcium (Ca) (Diehl et al., 2018), and the blood Ca level (Collazos et al., 2018; Lopera et al., 2018). Heat stress raises respiratory CO2 loss (respiratory alkalosis) and Na and K loss (coupled with bicarbonate ions) via elevated urine and sweat production. In principle, corrections in blood acid–base balance and associated electrolyte losses may be performed through modification of the DCAD. Diets formulated with DCAD of 200 to 370 mEq/kg DM have been recommended for optimal milk yield in lactating dairy cattle (West et al., 1991), 250 mEq/kg DM for optimal growth in chickens (Mongin, 1981) and pigs (Patience et al. 1987). In ruminants, dietary changes of this nature may directly modify ruminal pH, with associated effects on digestion, DMI, and ruminal microbial efficiency. Modifications in dietary salt concentrations to change DCAD may also directly affect diet digestibility and DMI. The effect of DCAD changes on the performance of sheep has received limited attention. Therefore, the role of DCAD on organic matter digestibility, DMI, digestibility, body weight, blood metabolites, and carcass traits of male lambs has not been directly assessed. Therefore, this study aimed to examine the effects of increasing DCAD from +150 to +750 mEq/kg of DM on organic matter digestibility, DMI, body weight, blood metabolites, and carcass traits in producing male lambs.

Furthermore, we conducted this study to explore whether positive DCAD in a high-temperature environment can improve the DMI, production, and carcass traits of lambs.

## MATERIALS AND METHODS

### Animals and Management

Animals were bred at Islamic Azad University scientific farm from June 2020 to October 2021 and approved by the Islamic Azad University Committee of Experimental Animal Ethics with the number code IU-2020-P016. Using a completely randomized block design, 40 Zandi male lambs (a native sheep breed in the central regions of Iran) with similar body weights (BW; 39.06 kg, SD = 0.55) were blocked to five treatments of eight replicates with 1 lamb per replicate. Lambs were fed in their individual cages during the whole experiment. Animals were fed with varying DCAD levels (mEq/kg DM): +150 (group 1 or Control group), +300 (group 2), +450 (group 3), +600 (group 4), and +700 (group 5). The diet was pelleted as TMR (total mixture ration) with a ratio of concentrate to roughage at 40:60. The sodium bicarbonate (NaHCO3) and sodium carbonate (Na+CO+) were included to increase DCAD. The experiment duration was 100 d, including a 21-d adaption period and a 79-d trial period. After that, in the trial period, lambs were fed the treatment diets at 0900 and 1800 hours. All lambs had free access to water during the experimental process. Ingredients and chemical components of diets are shown in Table 1.

**Table 1.** Ingredients and chemical components of diet for lambs (diets were formulated according to the recommendations of NRC (2001) and gradually provided to lambs. This experiment included five diets with the same energy and protein levels)

Items, %	Group 1, +150 mEq/kg DM	Group 2, +300 mEq/kg DM	Group 3, +450 mEq/kg DM	Group 4, +600 mEq/kg DM	Group 5, +750 mEq/kg DM
	40.00	40.00	40.00	40.00	40.00
Wheat straw	3.87	3.66	3.45	3.24	3.03
Soybean meal	19.62	19.62	19.62	19.62	19.62
Barley	7.69	7.69	7.69	7.69	7.69
Corn meal	25.46	25.46	25.46	25.46	25.46
Rice bran	2.25	2.25	2.25	2.25	2.25
Salt	0.90	0.90	0.90	0.90	0.90
NaHCO_3_	0.07	0.14	0.21	0.28	0.35
K_2_CO_3_	0.14	0.28	0.42	0.56	0.70
Analyzed nutrient composition, %					
DM	45.69	45.49	45.57	44.88	44.45
CP	16.68	16.84	16.91	16.94	16.74
Ash	6.36	6.42	6.55	6.64	6.75
OM	93.64	93.58	93.45	93.36	93.24
ADF	30.75	30.64	30.52	30.44	30.41
NDF	39.94	39.75	39.52	39.44	39.32
Na, mEq/100g DM	4.48	5.00	5.64	6.01	7.00
K, mEq/100g DM	36.78	37.10	42.10	44.10	47.10
Cl, mEq/100g DM	10.57	8.71	8.01	7.72	6.88
S, mEq/100g DM	7.87	8.31	8.73	8.11	9.12

DCAD, dietary cation–anion difference; CHOL, cholesterol; Na, sodium, K, potassium; Mg, magnesium; P, phosphorus. K, potassium; Mg, magnesium; P, phosphorus.

### Temperature-Humidity Index (THI) monitoring and measurement of rectal temperature

Two wet and dry-bulb thermometers were hung on the feeding barns at 1.5 m above the floor to ensure necessary ventilation and be away from sunlight and rain. The National Research Council (1971) then used the following formula to determine the THI:


THI=(Td+Tw)x0.72+40.6

Td and Tw are the temperature readings on the dry-bulb and wet-bulb thermometers. The daily averages of THI, Td and Tw were calculated. The rectum (about 4 to 6 cm from the annas) temperature (RT) of each lamb was recorded using an electronic thermometer at 0800, 1400, and 1800 hours every day during the experimental period.

### Data Collection and Determination of Digestibility

Diet samples were collected daily for 21 to 100 d and composited, dried at 65 ℃, and pulverized to pass a 1-mm screen for proximate chemical composition measurement of DM, crude protein (CP), crude ash, and total fat (AOAC, 1990), neutral detergent fiber (NDF), and acid detergent fiber (ADF) (Van Soest et al., 1991). Determine dry matter intake (DMI) was done according to National Research Council (1971). An atomic absorption spectrophotometer (iCE3000 SERIES, Thermo Fisher Scientific, USA) measured the contents of Na, and K. Cl content was determined using silver nitrate titration. As previously described, the magnesium nitrate method determined the S level (Wang & Beede, 1990). According to Block (1984), the DCAD was determined using the following equation:


$$\begin{array}{l} DCAD=Na\left(\%\right)/0.0023+K\left(\%\right)/0.0039\\ \;-Cl\left(\%\right)/0.00355-S\left(\%\right)/0.0016 \end{array}$$

All lambs were weighed individually every 14 d for 100 d. The DMI was recorded daily for each lamb calculated by the allowance of refusals.

### Carcass Composition

At the end of the experiment, after 16 h of food deprivation, the lambs were weighed and slaughtered, the weight of the warm carcass and collected blood were weighed, and then the scalp, legs, lungs, liver, spleen, kidneys, heart, testicles, and fat were weighed. The inside of the intestine and stomach (full and empty) were weighed. Carcass length was measured from the inner edge of the hip bone to the front part of the breastbone with a tape measure. The cross-sectional area of the rectus muscle (between the 12th and 13th ribs) was first drawn using oil paper and then measured with a digital area meter. A caliper measured back fat thickness between the 12th and 13th ribs. The carcasses of the lambs were divided into different parts of the neck, head, chest, throat, thigh, and tail, and the weight of each part was determined. The right half of the carcass of all the lambs was taken to the cold house and kept for 24 h at a temperature of 2 to 4 ℃, then each piece was separated by tissue, and the meat, fat (subcutaneous and intermuscular), and bone were separated from each piece. Separated and weighed (AOAC, 1999). In order to chemically analyze the components of the carcass (total fat and meat) obtained from the analysis of different parts of the right side of the carcass, they were mixed and ground, and the samples were analyzed according to the method suggested by AOAC.

### Rumen Fluid Collection and Volatile Fatty Acids Analysis

Rumen fluid samples were collected from each lamb using a stomach tube connected to a syringe. The rumen fluid pool used the double tubes method to avoid excessive saliva contamination. The outer rubber tube (i.d. = 2.5 cm) was specified to the mouth gag. The inner rubber tube (o.d. = 1.2 cm) was the collecting tube (110 cm) handed into the ruminal cavity. Approximately 25 mL of fluid samples were taken 2.5 h after morning feeding every day. The pH was immediately determined with a pH meter (pH221, Lutron, Taipei, Taiwan). After that, the ruminal fluid samples were filtered through two layers of cheesecloth, and 1 mL 6 N HCl was added for preservation. Then, samples were frozen at –20 °C for later analysis of osmolality, volatile fatty acids (VFAs), and NH3-N. The ruminal fluid osmolality was measured with an osmometer (Osmometer 3D3; Advanced Instruments Inc., Boston, MA, USA). The VFAs were prepared and analyzed as described by Thammacharoen et al. (2001). NH3–N was determined with a salicylate–hypochlorite method (Bower and Holm-Hansen, 1980).

### Measurement of Plasma Metabolites

Blood samples were collected monthly for analysis of biochemical parameters of blood serum. The samples were collected from the jugular vein and placed in EDTA vacuum tubes containing EDTA and intravenous vein. The pH of the samples was measured, and then the blood plasma was separated by centrifugation and frozen for further analysis. All samples were determined in terms of minerals such as sodium, potassium and magnesium, phosphorus, glucose, and blood cholesterol through the proposed AOAC methods.

### Statistical Analysis

Balances of energy and protein digestible in the small intestine were calculated for each lamb during each period according to Institut National de la Recherche Agronomique (1989). Intake, weight gain, blood metabolite, and composition, BW, digestibility, and ruminal fluid parameters, were analyzed using the mixed model procedure of SAS Institute (1990) according to a randomized complete block design was used for data analysis. This experiment is performed according to the following statistical model.


Yij=μ+Ti+Eij

In this regard, Yij is the value of each variable; µ is the mean of the related trait, Ti effect of treatment, and Eij error rate. And then LSD test method was used to compare the mean of each trait. Statistical significance was defined as *P* < 0.05.

## RESULTS

### THI and Rectal Temperature of Lambs

**Figure 1.**
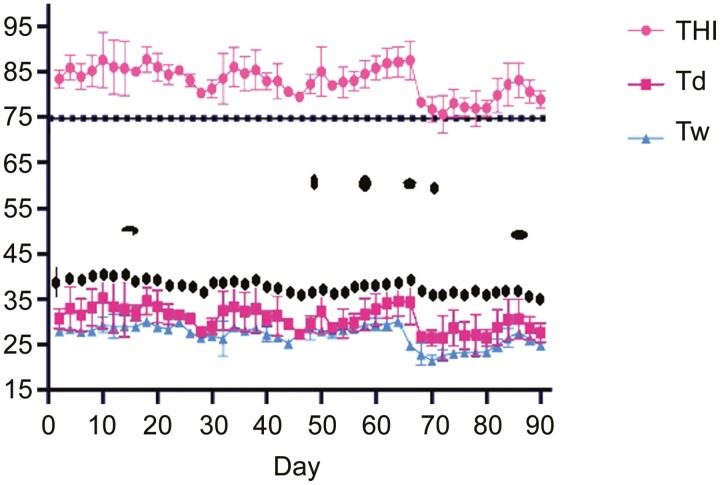
The daily average temperature-humidity index (THI), dry-bulb temperatures (Td) and wet-bulb temperatures (Tw) during the experimental period. The horizontal line at 75 indicates the threshold for heat stress.

The dry-bulb thermometer readings varied from 29.03 to 39.25 °C, and the mean for the period was 33.94 ± 2.5 °C (SD). The wet-bulb thermometer readings varied from 20.16 to 30.01, and the mean was 25.61 ± 2.24 °C. The calculated THI varied from 75.13 to 86.55, and the mean was 81.23 ± 3.35. During the entire experiment period, the THI was consistently higher than 75. There were no significant differences in rectal temperature between the control and other groups (Figure 1). There were no significant differences in the rectal temperature between DCAD groups (Table 2).

**Table 2.** Effects of dietary cation–anion difference (DCAD) on the rectal temperatures in male lambs

Items			Treatment			
	Group 1, +150 mEq/kg DM	Group 2, +300 mEq/kg DM	Group 3, +450 mEq/kg DM	Group 4, +600 mEq/kg DM	Group 5, +750 mEq/kg DM	*P*-value
Rectal temperature, °C	37.8	37.9	37.8	37.6	37.8	0.85

### DCAD Effect on Average Body Weight

The analysis of variance showed a statistically significant between the studied treatments in terms of average body weight of lambs in 28 to 42 d (*P* = 0.01). Comparing the weighting trait showed that the highest weighting was observed in group 4 (*P* = 0.02) (Table 3). However, this treatment did not have a statistically significant from other treatments in terms of increasing the average body weight of the lambs (groups 3 and 5). As well, the average body weight of the lambs receiving the treatments of the group 4 was 50.37 kg in the whole period; it was statistically higher than the other treatments.

**Table 3.** Effects of DCAD and average body weight of male lambs during each period (kg)

Period		Treatment				
	Group 1, +150 mEq/kg DM	Group 2, +300 mEq/kg DM	Group 3, +450 mEq/kg DM	Group 4, +600 mEq/kg DM	Group 5, +750 mEq/kg DM	*P*-value
0-14	36.32^b^	38.37^a,b^	38.06^a,b^	42.12^a^	41.12^a^	0.05
14-28	39.10^b^	41.01^a,b^	40.63^a,b^	45.00^a^	41.15^a,b^	0.02
28-42	40.31	42.29	42.79	47.07	42.56	0.24
42-56	45.82	45.66	46.46	49.15	47.60	0.75
56-70	48.75	49.37	48.68	51.61	51.02	0.84
70-84	50.21	55.50	50.91	54.45	53.06	0.06
84-98	51.36	55.50	53.81	55.95	55.26	0.86
98-112	52.36	58.00	56.08	57.66	55.26	0.66
Average	45.90^b^	47.81^a,b^	46.83^a,b^	50.37^a^	48.64^a,b^	0.00

### DCAD Effect on Carcass Parts

The results of analyzing the variance of the relative weight of the carcass and internal organs in the studied lambs are shown in Table 4. The results showed a statistical significance between the treatments in terms of live weight, carcass weight, carcass yield (*P* = 0.00), length and width of muscle cross section, lung weight, and abdominal fat (*P* = 0.05).

**Table 4.** Effect of varying dietary cation–anion difference on different parts of male lambs

Items		Treatment				
	Group 1, +150 mEq/kg DM	Group 2, +300 mEq/kg DM	Group 3, +450 mEq/kg DM	Group 4, +600 mEq/kg DM	Group 5, +750 mEq/kg DM	*P*-Value
**Live weight, kg**	61.54^b^	65.81^a^	66.90^a^	68.00^a^	66.51^a^	0.00
**Carcass weight, kg**	29.90^b^	33.63^a^	34.11^a^	35.98^a^	35.04^a^	0.00
** Carcass yield, %**	48.58^b^	51.10^a^	50.98^a^	52.91^a^	52.68^a^	0.00
** Blood weight, kg**	2.46	2.55	2.22	2.13	2.12	0.44
**Skin weight, kg**	3.44	3.10	3.53	3.01	3.83	0.24
** Head weight, kg**	2.60	2.31	2.10	2.04	2.04	0.75
**Leg weight, kg**	0.34	0.33	0.33	0.31	0.30	0.84
**Cross-sectional length of the muscle, cm**	13.24^a^	13.87^b^	13.24^c^	14.91^b^	14.41^b^	0.05
**Cross-sectional width of the muscle, cm**	5.51^ab^	5.42^b^	5.21^b^	5.95^a^	5.65^ab^	0.05
**Carcass length, cm**	112.24	112.00	111.50	112.23	112.00	0.32
** Lung weight, kg**	0.82^a^	0.61^c^	0.69^bc^	0.74^b^	0.84^a^	0.05
** Liver weight, kg**	0.36	0.38	0.39	0.40	0.41	0.77
** Spleen weight, kg**	0.16	0.18	0.19	0.19	0.20	0.07
** Kidney weight, kg**	0.15	0.16	0.16	0.17	0.17	0.82
** Heart weight, kg**	0.27	0.28	0.28	0.30	0.30	0.78
** Testis weight, kg**	0.10	0.11	0.11	0.12	0.11	0.89
**Tail weight, kg**	3.12	2.88	2.94	3.02	3.08	0.56
**Abdominal fat weight, kg**	2.77^a^	1.65^c^	1.80^c^	2.11^bc^	2.39^ab^	0.05

### Muscle

The effect of treatments on the cross-sectional area of the muscle in lambs is shown in Table 4. The results showed that the control group had the most significant effect on the cross-sectional length of lambs’ muscles. The effects of treatments on the width of the cross-sectional area are also shown in Table 4. The results showed that the maximum cross-sectional width of the muscle was observed in group 4. Also, the lowest muscle cross-sectional width in the studied lambs in group 3 (*P* = 0.05).

### Lung

The effect of treatments on the weight of the internal organs (lung) is shown in Table 4. The results showed that the highest weight of the lung was observed in group 5 (*P* = 0.05) and the control group. Also, the lowest lung weight was observed in group 2 (*P* = 0.05).

### Spleen

The effect of treatments on the weight of the internal organs (spleen) is shown in Table 4. The results showed that the highest spleen weight was observed in group 5. Also, the lowest spleen weight was observed in the control group. Although, there was no statistical differences between different treatments.

### Abdominal Fat

The effect of treatments on fat weight is shown in Table 4. The results showed that the highest fat weight was observed in the control group; however, this amount was not statistically significant from group 5. Also, the lowest weight of the fat was observed in group 2 (*P* = 0.05).

### Other Parts of the Carcass

The effect of treatments on the weight of the carcass and internal organs are shown in Table 4. The results showed no statistically significance between the studied groups in terms of blood weight, skin weight, head weight, leg weight, carcass length, liver weight, kidney weight, heart weight, testicle weight, tail weight, and rumen weight. However, numerically, the highest blood weight, skin weight, leg weight, carcass length, liver weight, testicle weight, tail weight, and rumen weight were observed in the control group. Also, the highest weight of kidney, heart, tail, and carcass length was observed in group 4.

### DCAD Effect on Dry Matter Intake and Digestibility

The mean comparison of treatments on DMI is shown in Table 5. The lowest DMI and CP digestibility were observed in the control group (*P* = 0.02). Furthermore, the highest DMI was observed in groups 5 and 4 (*P* = 0.01). Furthermore, the highest apparent digestibility of DMI, CP, and organic matter were observed in group 4 (*P* = 0.02). The lowest CP digestibility was observed in group 1 (control) (*P* = 0.02).

**Table 5.** Effects of dietary cation and anion difference on dry matter intake, organic matter digestibility and crude protein digestibility in lambs

Items		Treatment				
	Group 1, +150 mEq/kg DM	Group 2, +300 mEq/kg DM	Group 3, +450 mEq/kg DM	Group 4, +600 mEq/kg DM	Group 5, +750 mEq/kg DM	*P*-value
DMI, g/kg BW	31.60^b^	36.75^a^	38.11^a^	38.21^a^	37.11^a^	0.01
Nutrient intake, g/kg BW/d						
Organic matter	29.59^b^	34.39^a^	35.61^a^	35.67^a^	34.60^a^	0.04
** **Crude protein	4.44^b^	5.46^a^	6.53^a^	6.42^a^	6.83^a^	0.04
Neutral detergent fiber	16.60	16.90	19.10	19.07	19.04	0.09
** **Acid detergent fiber	8.34	8.56	9.33	9.59	9.30	0.84
Apparent digestibility, %						
Dry matter	73.74^b^	74.40^a^	77.10^a^	78.75^a^	80.01^a^	0.02
Organic matter	61.50^b^	68.05^a^	73.50^a^	74.86^a^	76.23^a^	0.02
** **Crude protein	65.87^b^	73.03^a^	77.05^a^	78.08^a^	78.22^a^	0.02

DCAD, dietary cation and anion difference; DMI, dry matter intake; BW, body weight; DM, dry matter.

### DCAD Effect on Rumen Fermentation Parameters

The highest ruminal pH was observed in the control group. As well, the lowest ruminal pH was observed in group 5 (*P* = 0.04). The variation of DCAD did not affect total VFAs, acetate, propionate, butyrate, valeric, isovaleric, acetate/propionate, urea nitrogen, and ammonia (Table 6). Nevertheless, numerically, the highest total VFAs, acetate, propionate, butyrate, valeric, isovaleric, acetate/propionate, urea nitrogen, and ammonia, were observed in the control group. Moreover, the lowest total VFAs, acetate, propionate, butyrate, valeric, isovaleric, acetate/propionate, urea nitrogen, and ammonia, were observed in group 5 (Table 6).

**Table 6.** Effects of DCAD on the rumen fermentation parameters of male lambs

Items		Treatment				
	Group 1, +150 mEq/kg DM	Group 2, +300 mEq/kg DM	Group 3, +450 mEq/kg DM	Group 4, +600 mEq/kg DM	Group 5, +750 mEq/kg DM	*P*-value
pH	6.54^b^	6.68^ab^	6.64^a,b^	6.71^a,b^	6.82^a^	0.04
Total VFA, mEq/L	75.15	72.21	71.36	72.65	73.40	0.33
Acetate, %	61.46	56.55	58.22	55.13	54.12	0.44
Propionate, %	19.44	19.10	18.53	18.01	17.83	0.24
Butyrate, %	13.60	13.31	13.10	13.04	13.04	0.75
Valeric, %	0.34	0.33	0.33	0.31	0.30	0.84
Isovaleric, %	1.14	0.96	0.98	0.84	1.01	0.06
A/P	3.74	3.20	3.10	3.08	3.01	0.86
Ammonia, mEq/L	112.50	112.00	111.50	111.00	110.23	0.32

A/P, acetic acid/propionic acid.

### DCAD Effect on Plasma Metabolites

The variance analysis results of blood biochemical parameters showed a significant difference between treatments in glucose, cholesterol, and sodium parameters in lambs (*P* = 0.04, Table 7). The results demonstrated that the control group had the highest effect on blood glucose levels in lambs (*P* = 0.04). Groups 3, 4, and 5 did not differ significantly in blood glucose levels. There was a significant difference between group 5 comparisons of the other group in blood cholesterol, with the highest effect on blood cholesterol. There were no significant differences between blood phosphorus, magnesium, and potassium levels (*P* = 0.05). Regardless, the highest amount of phosphorus, magnesium, and potassium in the blood was observed in group 5. The lowest levels of phosphorus, magnesium, and potassium in the blood were observed in the control group (Table 7). The highest amount of sodium in the blood was observed in group 1 (*P* = 04).

**Table 7.** Effect of dietary cation–anion difference on the plasma metabolites and nitrogen balance of male lambs

Items	Treatment					
	Group 1, +150 mEq/kg DM	Group 2, +300 mEq/kg DM	Group 3, +450 mEq/kg DM	Group 4, +600 mEq/kg DM	Group 5, +750 mEq/kg DM	*P*-value
Ca, mmol/L	2.82^a^	2.71^a,b^	2.64^a,b^	2.61^a,b^	2.42^b^	0.05
Glc, mmol/L	105.15^a^	104.21^b^	103.36^c^	103.55^c^	103.40^c^	0.04
CHOL, mmol/L	116.46^b^	116.55^b^	115.22^b^	115.13^b^	118.12^a^	0.05
P, mmol/L	1.45	1.56	1.57	2.05	2.17	0.75
Mg, mmol/L	1.13	1.00	1.76	1.30	1.76	0.82
K, mmol/L	113.39	113.30	113.44	113.50	113.52	0.84
Na, mmol/L	124^a^	108^a,b,c^	111^a,b^	94^b,c^	84^c^	0.04
Cl, mmol/L	112.50	112.00	111.50	111.00	110.23	0.32
Nitrogen balance, g/d	9.65	9.40	11.80	12.39	12.06	0.83

DCAD, dietary cation–anion difference; CHOL, cholesterol; Na, sodium, K, potassium; Mg, magnesium; P, phosphorus. K, potassium; Mg, magnesium; P, phosphorus.

## DISCUSSION

In the present study, we investigated the effect of the DCAD diet on the carcass traits, production performance, and digestibility in lambs under the environmental high temperatures. In this regard, Santos et al. (2019) and Lean et al. (2019) used Meta-analyzes to determine the variable effects of DCAD on cattle performance, production, and health. They concluded that prenatal DCAD negatively increases total blood calcium levels during labor and leads to fewer diseases. Our results revealed a statistical significance between treatments in ruminal pH. Based on the findings of this investigation, the kind of cation source used in the diet of lambs has a direct effect on the anion–cation balance of the diet; it can affect ruminal fermentation parameters. The concentration of ammonia nitrogen produced in the rumen is one of the indicators to study the conditions of rumen fermentation. A good diet provides the nitrogen needed for maximum microbial protein, prevents excess ammonia-related waste, and provides sufficient nondegradable protein. Ammonia is one of the nitrogenous compounds used by microbes in rumen to synthesize protein. Rumen ammonia is produced from nitrogenous substances in the diet, urea in saliva, and penetrating urea through the rumen wall (Mathieson and Milligan 1971). Since the concentration of ammonia decreases with increasing DCAD level, it can be stated that increasing DCAD probably increases the ruminal acidity. The decomposition of CP strongly influences the production of ammonia in the diet by micro-organisms and the decomposition of the microbial population due to nitrogen recycling under adverse conditions; it can be concluded that this increase in acidity balances the culture medium in favor of further synthesis. Dudareva et al. (2004) displayed that reducing rumen ammonia production is beneficial in increasing microbial protein production or reducing rumen protein breakdown. Doepel and Hayirli (2011) reported that adding sodium bicarbonate to the diet did not affect ruminal ammonia levels, which was consistent with the results of this study. Reducing or eliminating protozoa from the rumen prevents the energy cycle between bacteria and protozoa, which reduces the breakdown of bacterial proteins. As a result, the flow of microbial nitrogen from the rumen increases, and consequently, the concentration of ammonia decreases (Thanh et al. 2020). Harrison et al. (2012) reported that potassium carbonate could be used as an effective buffer to stabilize ruminal pH, increase DMI, increase volatile fatty acid production, and increase the acetate to propionate ratio, consistent with the results of this research. Shahzad et al. (2008) displayed that increasing the level of anion–cation difference in the diet maintains the fermentation pattern to produce balanced acetate and butyrate, which in turn increases the synthesis of fatty acids of domestic origin, which provides up to 25% of milk fat. Therefore, it is observed that increasing DCAD has a positive effect on reducing environmental pollution related to methane production, which is an important point in modern practical nutrition. Previous studies have shown that increasing DCAD via potassium carbonate increases DMI (Apper-Bossard et al. 2010). As well, the highest apparent dry matter digestibility and the area below the digestibility curve (fluctuations of dry matter digestibility at different times) are related to the treatment containing potassium carbonate and magnesium carbonate with DCAD 905 + mEq level; it can be concluded that the addition of potassium carbonate to dairy cows’ diet improves the rumen fermentation and provides potassium to the microbial population, increasing dry matter digestibility (West et al., 1987). Iwaniuk and Erdman (2015) also reported increased dry matter digestibility with increasing DCAD. Funk et al. (1986) investigated the effect of adding potassium with lasalocid on the digestibility of insoluble fiber in neutral detergent and concluded that the simultaneous use of the two increases the digestibility of insoluble fiber in neutral detergent. We showed that with increasing DCAD, ruminal acidity increased, which increased the digestion of insoluble fiber in a neutral detergent, followed by an increase in dry matter digestibility, which was supported by the previous study (Martinez et al. 2018). Furthermore, blood measurements are useful to reflect the metabolic status of animals. Because blood glucose concentration in ruminants is more affected by the amount of ruminal fluid propionate (McDonald et al. 2010). Therefore, various factors such as diet, source, and amount of supplementation that affect ruminal propionate production (Spears et al. 2004) can change the blood parameters. Minor changes in plasma sodium and potassium may be attributed to dietary changes in these minerals because excess sodium and potassium are excreted by the kidneys (Hu and Murphy 2004). Similar results were reported by West et al. (1991), who stated that increasing the level of DCAD (−116 to + 312 mA/kg DM) had no significant effect on plasma sodium and potassium levels.

Furthermore, this study showed a significant difference between the treatments in terms of average body weight of lambs in 28 to 42 d. As well, the average body weight of the lambs receiving the treatments of the group 4 was 50.37 kg in the whole period; it was significantly higher than the other treatments. In agreement with the results of the present study, the use of different levels of organic and inorganic supplements in fattening calves caused an increase in BW and a difference in feed intake (Malcolm-Callis et al., 2000). Also, in another experiment, feeding fattening lambs with 20 mg of organic and mineral supplements reduced feed consumption (Garg et al., 2008). Despite this, some other studies reported the lack of effect of mineral and organic supplements on feed consumption. The decrease in feed consumption in lambs receiving supplements in the present study can be caused by various factors. It seems that other factors, such as the decrease in some mineral of the diet, caused the decrease in feed consumption of lambs receiving sodium bicarbonate and carbonate supplements. The use of supplements improved the efficiency of using nutrients to increase the weight of lambs in the present study. Also, the lambs consumed more than three percent of the average live BW of the feed. Therefore, saving in the use of nutrients and improving the efficiency of their use can be one of the possible factors for reducing feed consumption in this research. On the other hand, other factors, such as improving the absorption of nutrients (Yari et al., 2010) and increasing the concentration of VFAs in the blood (Malcolm-Callis et al., 2000), can also reduce feed consumption in ruminant animals. This study also showed a significant difference between the related treatments, including dry matter intake (DMI), organic matter digestibility, and CP. The increase in digestibility may refer to the lack of the rumen microbial population requirements in the basic diet (Mallaki et al., 2015). Some factors such as supplement concentration, supplement source (organic or mineral), diet balance (ratio of forage to concentrate), and base diet supplement concentration were the factors that differentiated nutrient digestibility in different studies (Garg et al., 2008; Kun et al., 2015).

Our results also showed that different DCAD treatments affected some carcass parts’ weight. These results were consistent with the studies by Luebbe et al. (2011) and Schoonmaker et al. (2013), in which they studied 16 mEq/100 g DM DCAD diets for a long (145 and 196 d) and a short (14 d) period, respectively. As well, Ross et al. (1994) reported that carcass marbling score increased by consuming cationic diets for 84 d. Schoonmaker et al. (2013) suggested that an increase in marbling of cattle fed positive DCAD could be a result of increased production of volatile fatty acid in the rumen due to the ability of sodium bicarbonate to increase ruminal pH and enhance fiber digestibility. These might have been contra-indicative to overall productivity such as carcass weight or marbling deposition if the steers had been fed a negative DCAD diet for a longer period. Therefore, various factors such as diet, source, and supplementation affect product performance. However, further studies and experiments on the effect of DCAD on carcass traits will be needed.

In conclusion, we present the associated carcass traits, production performance, and digestibility mechanisms that contribute to the effect of the DCAD diet on lambs under heat-stress conditions. The results showed a significant difference between treatments regarding DMI and protein digestibility, ruminal pH, some blood metabolites, and carcass parts of lambs during 100 d. These results may provide the feasibility of feeding various DCADs to male lambs.

## References

[CIT0001] Al-Rabadi, G., and M. Al-Hijazeen. 2018. Variation in dietary cation-anion differences (DCAD) of feed ingredients in relation to milk fever disease in dairy cattle. Ukr. J. Ecol. 8:51–56. doi:10.15421/2018_186

[CIT0002] AOAC. 1999. Official Methods of Analysis of AOAC International. 16th ed. 5th rev. ed. P. Cunniff, ed. Gaithersburg, MD: AOAC Int.

[CIT0003] Apper-Bossard, E., P. Faverdin, F. Meschy, and J. L. Peyraud. 2010. Effects of dietary cation-anion difference on ruminal metabolism and blood acid-base regulation in dairy cows receiving 2 contrasting levels of concentrate in diets. J. Dairy Sci. 93:4196–4210. doi:10.3168/jds.2009-2975 20723694

[CIT0004] Block, E. 1984. Manipulating dietary anions and cations for prepartum dairy cows to reduce incidence of milk fever. J. Dairy Sci. 67:2939–2948. doi:10.3168/jds.S0022-0302(84)81657-4 6530489

[CIT0005] Bower, C. E., and T. Holm-Hansen. 1980. A salicylate–hypochlorite method for determining ammonia in seawater. Can. J. Fish Aqua Sci. 37:794–798. doi:10.1139/f80-106

[CIT0006] Collazos, C., C. Lopera, J. E. P. Santos, and J. Laporta. 2018. Effects of the level and duration of maternal diets with negative dietary cation-anion differences prepartum on calf growth, immunity, and mineral and energy metabolism. J. Dairy Sci. 100:9835–9850. doi:10.3168/jds.2017-13200 28987581

[CIT0007] Diehl, A. L., J. K. Bernard, S. Tao, T. N. Smith, D. J. Kirk, D. J. McLean, and J. D. Chapman. 2018. Effect of varying prepartum dietary cation-anion difference and calcium concentration on postpartum mineral and metabolite status and milk production of multiparous cows. J. Dairy Sci. 101:9915–9925. doi:10.3168/jds.2018-14828 30219430

[CIT0008] Doepel, L., and A. Hayirli. 2011. Exclusion of dietary sodium bicarbonate from a wheatbased diet: Effects on milk production and ruminal fermentation. J. Dairy Sci. 94:370–375. doi:10.3168/jds.2010-3488 21183047

[CIT0009] Dudareva, N., E. Pichersky, and J. Gershenzon. 2004. Biochemistry of plant volatiles. Plant Physiol. 135:1893–1902. doi:10.1104/pp.104.049981 15326281 PMC520761

[CIT0010] Funk, M. A., M. L. Galyean, and T. T. Ross. 1986. Potassium and lasalocid effects on performance and digestion in lambs. J. Anim. Sci. 63:685–691. doi:10.2527/jas1986.633685x 3759698

[CIT0011] Garg, A. K., M. Vishal, and R. S. Dass. 2008. Effect of organic zinc supplementation on growth, nutrient utilization and mineral profile in lambs. Anim. Feed Sci. Technol. 144:82–96. doi:10.1016/j.anifeedsci.2007.10.003

[CIT0012] Harrison, J., R. White, R. Kincaid, E. Block, J. Jenkins, and N. St-Pierre. 2012. Effectiveness of potassium carbonate sesquihydrate to increase dietary cation-anion difference in early lactation cows. J. Dairy Sci. 95:3919–3925. doi:10.3168/jds.2011-4840 22720946

[CIT0013] Hu, W., and M. R. Murphy. 2004. Dietary cation-anion difference effects on performance and acid-base status of lactating dairy cows. A meta-analysis. J. Dairy Sci. 87:2222–2229. doi:10.3168/jds.S0022-0302(04)70042-9 15328236

[CIT0014] Iwaniuk, M. E., and R. A. Erdman. 2015. Intake, milk production, ruminal, and feed efficiency responses to dietary cation-anion difference by lactating dairy cows. J. Dairy Sci. 98:8973–8985. doi:10.3168/jds.2015-9949 26409960

[CIT0015] Kun, B., S. Weili, L. Chunyi, W. Kaiying, L. Zhipeng, B. Shidan, and L. Guangyu. 2015. Effects of dietary zinc supplementation on nutrient digestibility, haematological biochemical parameters and production performance in male Sika deer (Cervus nippon). Anim. Prod. Sci. 56:997–1001. doi:10.1071/AN15039

[CIT0016] Lean, I. J., J. E. P. Santos, E. Block, and H. M. Golder. 2019. Effects of prepartum dietary cation-anion difference intake on production and health of dairy cows: A meta-analysis. J. Dairy Sci. 102:2103–2133. doi:10.3168/jds.2018-14769 30594362

[CIT0017] Leno, B., C. Ryan, T. Stokol, D. Kirk, K. Zanzalari, J. Chapman, and T. Overton. 2017. Effects of prepartum dietary cation-anion difference on aspects of peripartum mineral and energy metabolism and performance of multiparous Holstein cows. J. Dairy Sci. 100:4604–4622. doi:10.3168/jds.2016-12221 28434740

[CIT0018] Lopera, C., R. Zimpel, A. Vieira-Neto, F. R. Lopes, W. Ortiz, M. Poindexter, B. N. Faria, M. L. Gambarini, E. Block, C. D. Nelson, et al. 2018. Effects of level of dietary cation-anion difference and duration of prepartum feeding on performance and metabolism of dairy cows. J. Dairy Sci. 101:7907–7929. doi:10.3168/jds.2018-14580 29885896

[CIT0019] Luebbe, M. K., G. E. Erickson, T. J. Klopfenstein, M. A. Greenquist, and J. R. Benton. 2011. Effect of dietary cation-anion difference on urinary pH, feedlot performance, nitrogen mass balance, and manure pH in open feedlot pens. J. Anim. Sci. 89:489–500. doi:10.2527/jas.2009-2458 21262979

[CIT0020] Mallaki, M., M. A. Norouzian, and A. A. Khadem. 2015. Effect of organic zinc supplementation on growth, nutrient utilization, and plasma zinc status in lambs. Turkish J. Vet. Anim. Sci. 39:75–80. doi:10.3906/vet-1405-79

[CIT0021] Martinez, N., R. M. Rodney, E. Block, L. L. Hernandez, C. D. Nelson, I. J. Lean, and J. E. P. Santos. 2018a. Effects of prepartum dietary cation-anion difference and source of vitamin D in dairy cows: Lactation performance and energy metabolism. J. Dairy Sci. 101:2544–2562. doi:10.3168/jds.2017-13739 29274965

[CIT0022] Martinez, N., R. M. Rodney, E. Block, L. L. Hernandez, C. D. Nelson, I. J. Lean, and J. E. P. Santos. 2018b. Effects of prepartum dietary cation-anion difference and source of vitamin D in dairy cows: Health and reproductive responses. J. Dairy Sci. 101:2563–2578. doi:10.3168/jds.2017-13740 29274983

[CIT0023] Mathieson, G. W., and L. P. Milligan. 1971. Nitrogen metabolism in sheep. Br. J. Nutr. 25:351–366. doi:10.1079/BJN19710100 5575210

[CIT0024] McDonald, P., R. A. Edwards, J. F. D. Greenhalgh, C.A. Morgan, L. A. Sinclair, R. G. Wilkinson. 2010. Animal nutrition. 7th ed. New York, USA: Longman Scientific and Technical; 692.

[CIT0025] Melendez, P., and S. Poock. 2017. A dairy herd case investigation with very low dietary cation-anion difference in prepartum dairy cows. Front. Nutr. 4:26. doi:10.3389/fnut.2017.00026 28660195 PMC5468380

[CIT0026] Mongin, P. 1981. Recent advances in dietary anion–cation balance: applications in poultry. Proc. Nutr. Sac. 40:285–294. doi:10.1079/pns19810045 7301833

[CIT0027] National Research Council. 1971. A guide to environmental research on animals. In: National Academies Press, Washington, DC: National Academies Science.

[CIT0028] Patience, J. F., R. E. Austic, and R. D. Boyd. 1987. Effect of dietary electrolyte balance on growth and acid–base status in swine. J. Anim. Sci. 64:457–466. doi:10.2527/jas1987.642457x 3030993

[CIT0029] Rodney, R., N. Martinez, E. Block, L. Hernandez, P. Celi, C. Nelson, J. Santos, and I. Lean. 2018a. Effects of prepartum dietary cation-anion difference and source of vitamin D in dairy cows: Vitamin D, mineral, and bone metabolism. J. Dairy Sci. 101:2519–2543. doi:10.3168/jds.2017-13737 29274979

[CIT0030] Rodney, R. M., N. P. Martinez, P. Celi, E. Block, P. C. Thomson, G. Wijffels, D. R. Fraser, J. E. P. Santos, and I. J. Lean. 2018b. Associations between bone and energy metabolism in cows fed diets differing in level of dietary cation-anion difference and supplemented with cholecalciferol or calcidiol. J. Dairy Sci. 101:6581–6601. doi:10.3168/jds.2017-14033 29655559

[CIT0031] Ross, J. G., J. W. Spears, and J. D. Garlich. 1994. Dietary electrolyte balance effects on performance and metabolic characteristics in finishing steers. J. Anim. Sci. 72:1600–1607. doi:10.2527/1994.7261600x 8071186

[CIT0032] Santos, J. E. P., I. J. Lean, H. M. Golder, and E. Block. 2019. Meta-analysis of the effects of prepartum dietary cation-anion difference on performance and health of dairy cows. J. Dairy Sci. 102:2134–2154. doi:10.3168/jds.2018-14628 30612801

[CIT0033] SAS Institute Inc. 1990. The SAS System of Windows, Release 5.0, SASI, Cary, NC, USA.

[CIT0034] Schoonmaker, J. P., K. T. Korn, K. N. Condron, C. N. Shee, M. C. Claeys, T. D. Nennich, and R. P. Lemenager. 2013. Effect of decreasing dietary cation anion difference on feedlot performance, carcass characteristics, and beef tenderness. J. Anim. Sci. 91:5762–5768. doi:10.2527/jas.2013-6525 24146160

[CIT0035] Shahzad, M. A., and M. Sarwar. 2008. Influence of altering dietary cation anion difference on milk yield and its composition by early lactating Nili Ravi buffaloes in summer. Livest. Sci. 113:133–143. doi:10.1016/j.livsci.2007.03.002

[CIT0036] Slater, C. J., E. L. Endres, S. R. Weaver, A. A. Cheng, M. R. Lauber, S. F. Endres, E. Olstad, A. DeBruin, P. M. Crump, K. E. Bloc, et al. 2018. Interaction of 5-hydroxy-l-tryptophan and negative dietary cation-anion difference on calcium homeostasis in multiparous peripartum dairy cows. J. Dairy Sci. 101:5486–5501. doi:10.3168/jds.2017-13938 29605319

[CIT0037] Spears, J. W., P. Schlegel, M. C. Seal, and K. E. Lloyd. 2004. Bioavailability of zinc from zinc sulfate and different organic zinc sources and their effects on ruminal volatile fatty acid proportions. Livest. Prod. Sci. 90:211–217. doi:10.1016/j.livprodsci.2004.05.001

[CIT0038] Thammacharoen, S., S. Chanpongsang, and N. Chaiyabutr. 2001. Effects of monensin administation on mammary function in late lactating crossbred Holstein cattle. Asian-Australas. J. Anim. Sci. 14:1712–1718. doi:10.5713/ajas.2001.1712

[CIT0039] Thanh, T. N., P. D. Van, T. D. Cong, T. Le Minh, and Q. H. N. Vu. 2020. Assessment of testis histopathological changes and spermatogenesis in male mice exposed to chronic scrotal heat stress. J. Anim. Behav. Biometeorol. 8:174–180. doi:10.31893/jabb.20023

[CIT0040] Tucker, W. B., G. A. Harrison, and R. W. Hemken. 1988. Influence of dietary cation–anion balance on milk, blood, urine, and rumen fluid in lactating dairy cattle. J. Dairy Sci. 71:346–354. doi:10.3168/jds.S0022-0302(88)79563-6 3379168

[CIT0041] Van Soest, P. J., J. B. Robertson, and B. A. Lewis. 1991. Methods for dietary fiber, neutral detergentfiber, and nonstarch polysaccharides in relation to animal nutrition. J. Dairy Sci. 74:3583–3597. doi:10.3168/jds.S0022-0302(91)78551-2 1660498

[CIT0042] Wang, C., and D. K. Beede. 1990. Effects of supplemental protein on acid-base status and calcium metabolism of nonlactating Jersey cows. J. Dairy Sci. 73:3178–3186. doi:10.3168/jds.S0022-0302(90)79008-X 2125606

[CIT0043] West, J. W., C. E. Coppock, D. H. Nave, J. M. Labore, L. W. Greene, and T. W. Odom. 1987. Effects of Potassium Carbonate and Sodium Bicarbonate on Rumen Function in Lactating Holstein Cows1. J. Dairy Sci. 70:81–90. doi:10.3168/jds.S0022-0302(87)79982-2 3033037

[CIT0044] West, J. W., B. G. Mullinix, and T. G. Sandifer. 1991. Changing dietary electrolyte balance for dairy cows in cool and hot environments. J. Dairy Sci. 74:1662–1674. doi:10.3168/jds.S0022-0302(91)78329-X 1908868

[CIT0045] Yari, L., M. Aghaalikani, and F. Khazaei. 2010. Effect of seed priming duration and temperature on seed germination behavior of bread wheat (Triticum aestivum L.). J. Agri. Biol. Sci. 5:7–15.

